# Sustainable Green Polymer Production for Pharmaceutical Manufacturing: A Review of Environmental and Economic Impacts

**DOI:** 10.3390/polym18070842

**Published:** 2026-03-30

**Authors:** Youssef Basem, Alamer Ata, Fayek Sabry, Maria Tamer, Elaria Raaft, Rehab Abdelmonem

**Affiliations:** 1 Medical and Pharmaceutical Industrial Biotechnology Department, College of Biotechnology, Misr University for Science and Technology (MUST), 6th of October City 12566, Giza, Egypt; 200025070@must.edu.eg (A.A.); 200023335@must.edu.eg (F.S.); 200030466@must.edu.eg (M.T.); 200028430@must.edu.eg (E.R.); 2 Industrial Pharmacy Department, College of Pharmaceutical Sciences and Drug Manufacturing, Misr University for Science and Technology (MUST), 6th of October City 12566, Giza, Egypt

**Keywords:** sustainable polymers, green polymer synthesis, pharmaceutical manufacturing, green chemistry, biopolymers, biodegradable polymers, life cycle assessment (LCA)

## Abstract

Polymers are fundamental components of modern pharmaceutical manufacturing, serving critical roles as excipients, binders, coatings, and matrices for controlled drug delivery systems. However, the conventional production of pharmaceutical polymers relies heavily on petrochemical feedstocks, energy-intensive processes, and hazardous solvents, leading to significant environmental and economic burdens. In recent years, increasing regulatory pressure, environmental awareness, and sustainability goals have driven the pharmaceutical industry toward greener manufacturing strategies. This review critically examines sustainable green polymer production for pharmaceutical applications, with a focus on both environmental and economic impacts. The review discusses the role of polymers in pharmaceutical manufacturing, outlines the limitations of conventional polymer synthesis, and highlights the relevance of green chemistry principles in addressing these challenges. Key green polymer synthesis techniques, including biopolymer production, enzymatic polymerization, microwave-assisted synthesis, supercritical CO_2_ processing, and the use of ionic liquids and deep eutectic solvents, are systematically evaluated. Additionally, life-cycle assessment (LCA) approaches are explored to assess the environmental performance of green polymer processes in comparison with traditional methods. Beyond environmental sustainability, this review emphasizes the importance of pharmacoeconomic evaluation in determining the feasibility of adopting green polymers at an industrial scale. Cost–benefit analyses, manufacturing cost comparisons, long-term economic advantages, and health–economic outcomes are discussed in the context of pharmaceutical supply chains. Regulatory perspectives, industrial implementation challenges, and future directions are also addressed. Overall, this review highlights sustainable polymer innovation as a critical pathway toward environmentally responsible, economically viable, and future-ready pharmaceutical manufacturing.

## 1. Introduction

Polymers play a central role in pharmaceutical manufacturing, being used not only as excipients in tablets, capsules, and injectable formulations but also as the foundational materials for advanced delivery systems such as nanoparticles, hydrogels, and implants that enable controlled drug release and targeted therapy. Their physical and chemical properties govern drug solubility, stability, release kinetics, and bioavailability, making polymer selection critical to therapeutic efficacy and patient outcomes. Beyond drug delivery, polymers serve as biocompatible scaffolds in tissue engineering and as materials for medical devices such as biodegradable sutures, orthopedic implants, and scaffolds for regenerative medicine. These applications make polymers indispensable to modern pharmaceutics and biomedical technologies [1].

Traditionally, the pharmaceutical industry has relied heavily on petrochemically derived polymers, such as polyethylene glycol (PEG), poly (methyl methacrylate) (PMMA), and polyvinylpyrrolidone (PVP), due to their well-characterized performance and ease of manufacture. However, these materials are associated with significant environmental burdens, including dependence on finite fossil resources, high energy demand in production, and persistence in the environment after disposal. The widespread use of non-biodegradable polymers contributes to plastic pollution and lifecycle greenhouse gas emissions, raising sustainability concerns among regulators, manufacturers, and the public. This has created an imperative to rethink polymer platforms in pharmaceutical contexts to reduce their environmental footprint without compromising material performance or patient safety [2].

The growing demand for sustainable manufacturing in pharmaceuticals reflects broader trends across the chemical and materials sectors. Stakeholders increasingly expect products and processes that align with environmental stewardship and circular economy principles, emphasizing resource efficiency, biodegradability, and recyclability throughout the product lifecycle. In polymer science, this shift has catalyzed research into bio-based and biodegradable polymers derived from renewable feedstocks such as plant biomass, agricultural residues, and microbial fermentation products. These materials include polylactic acid (PLA), polyhydroxyalkanoates (PHAs), polybutylene succinate (PBS), and other novel polymer families that offer the promise of reduced environmental impact and end-of-life degradability [3].

The environmental impact of conventional polymer synthesis is multifaceted, involving resource depletion, energy consumption, and persistent waste generation. Petrochemical feedstocks require energy-intensive extraction and refining, and the resulting polymers often resist degradation, accumulating in landfills and ecosystems. Moreover, solvent-based synthesis methods can produce hazardous by-products, further exacerbating ecological harm. These issues underscore the unsustainability of traditional polymer supply chains and highlight the need for new materials that integrate environmental considerations from synthesis to disposal [4].

In response, the field of polymer chemistry has embraced green chemistry principles, which advocate designing materials and processes that minimize waste, avoid hazardous substances, and maximize resource efficiency. Applied to polymer production, green chemistry encourages the use of renewable monomers, catalysts that reduce energy requirements, solvent-free or benign solvent systems, and strategies such as mechanochemistry and solvent-less polymerizations that further reduce environmental impacts. By prioritizing circularity and degradability, green chemistry aims to create polymers that can enter recovery and recycling streams or biodegrade safely at the end of life, thus reducing the ecological footprint of material use [4].

Bio-based polymers have emerged as a major focus of sustainable materials research. Derived from biological sources such as plant carbohydrates, microbial lipids, or fermentation products, these polymers often exhibit enhanced biodegradability and lower carbon intensity than their petroleum-derived counterparts. Materials such as PLA and PHAs have seen increasing adoption across industries, and their potential use in pharmaceutical applications, particularly for biodegradable drug delivery systems and biodegradable implants, is under active investigation. However, challenges remain, including variability in mechanical properties, scalability of production, and ensuring consistent quality for regulated pharmaceutical use [5].

Biodegradable biobased polymers not only reduce reliance on fossil resources but also support life-cycle environmental benefits, as their end-of-life degradation can convert them into benign compounds such as CO_2_, water, and biomass under appropriate conditions. Life-cycle assessments (LCAs) indicate that bio-based polymer production can reduce greenhouse gas emissions and energy consumption when compared to conventional plastics, provided that feedstock production and processing are managed sustainably. In pharmaceutical manufacturing, where materials are subject to stringent regulatory and performance requirements, integrating LCA perspectives helps stakeholders balance environmental impacts with clinical and economic performance [1].

The integration of sustainability into polymer innovation also intersects with economic considerations. Sustainable polymers often face barriers such as higher raw material costs, processing challenges, and uncertain market demand. Economic evaluations, including techno-economic analysis (TEA) and cost–benefit assessments, are essential to determine whether green polymer technologies can compete with incumbent materials at a commercial scale. Such analyses examine feedstock costs, processing efficiencies, waste management savings, and potential regulatory incentives that could enhance the economic attractiveness of sustainable materials [1].

In pharmaceutical contexts, where cost pressures and regulatory oversight are significant, the adoption of sustainable polymers necessitates evidence of both environmental and economic value. This encompasses not only material performance and biodegradability but also integration with existing manufacturing processes, compliance with pharmacopeial standards, and long-term cost savings from reduced waste handling and environmental liabilities. By combining green chemistry, life cycle thinking, and economic analysis, researchers and industry can guide the development of polymers that are both environmentally responsible and economically viable for pharmaceutical manufacturing [5].

Overall, the transition toward sustainable green polymer production represents a strategic opportunity for the pharmaceutical industry to align material innovation with environmental goals, regulatory expectations, and economic imperatives, laying the groundwork for next-generation materials that support both human health and planetary health [3].

## 2. Methodology

This review aims to critically evaluate recent advances in sustainable green polymer production for pharmaceutical manufacturing, with a particular focus on environmental and economic implications. The purpose is to synthesize current knowledge on green polymer synthesis techniques, assess their advantages and limitations compared with conventional petrochemical-based approaches, and identify key research gaps and future perspectives that can support the transition toward more sustainable pharmaceutical systems. The scope of this review encompasses polymers used in pharmaceutical applications, including excipients, coatings, controlled drug delivery matrices, hydrogels, and biodegradable systems, covering both traditional polymers and emerging bio-based alternatives derived from renewable resources. It further includes green synthesis approaches such as enzymatic polymerization, microwave-assisted synthesis, supercritical CO_2_ processing, and ionic liquid or deep eutectic solvent-based methods, in addition to life-cycle assessment (LCA), environmental sustainability metrics, pharmacoeconomic considerations, and regulatory perspectives to provide a comprehensive and integrated understanding of the field. The literature included in this review was selected based on predefined criteria, including relevance to green polymer synthesis and pharmaceutical applications, publication in peer-reviewed journals, and a primary focus on recent studies (2020–2025) to ensure up-to-date coverage The literature search was conducted using major scientific databases, including PubMed, Scopus, and Web of Science, employing keywords such as “green polymer synthesis,” “sustainable polymers,” “pharmaceutical polymers,” “biodegradable polymers,” and “green chemistry approaches.” The selection process involved initial screening based on titles and abstracts, followed by full-text evaluation to ensure relevance to the scope of this review. Although this study does not follow a formal systematic review protocol, efforts were made to ensure balanced coverage and critical selection of high-quality and recent publications. Emphasis was placed on studies providing experimental validation, comparative analyses, or clear sustainability metrics. Studies addressing environmental impact, sustainability performance, economic feasibility, and pharmaceutical functionality were prioritized, while non-relevant or non-pharmaceutical-focused works were excluded to maintain scientific rigor. This work is designed as a narrative critical review rather than a systematic review or meta-analysis, allowing for qualitative synthesis, comparative discussion, and critical interpretation of existing literature, with the objective of highlighting current trends, limitations, and future opportunities at the interface of green chemistry, polymer science, and pharmaceutical manufacturing.

## 3. Polymers in Pharmaceutical Manufacturing

Polymers play a central role in modern pharmaceutical manufacturing due to their versatility and tunable physicochemical properties. When it comes to chemicals, polymers are known for being high molecular weight. Polymers are made up of many smaller parts called monomers. These monomers are linked together by covalent bonds or other chemical reactions. When several monomer units are joined together to make a long chain polymer, this is called polymerization. Polymer nanoparticles (PNPs) are made using physical methods or direct nanosynthesis. They are then polymerized in micro- or nanoemulsions with nanoreactor sections. Polymers from both natural and man-made sources are widely used in the medicinal and biomedical fields, and their use is growing quickly. Polymers are used a lot in the pharmacy business today to control how drugs are released. Other applications of polymers are packaging materials, medical equipment, and packaging aids for pharmaceuticals, such as coating agents, suspending agents, emulsifying agents, adjuvants and adhesives [6].

### 3.1. Types of Polymers Used (Binders, Coating, Controlled Release Matrices, Excipients)

Depending on their functional role within the dosage form, pharmaceutical polymers can be categorized as follows:

#### 3.1.1. Binders and Excipients

**Figure 1:**
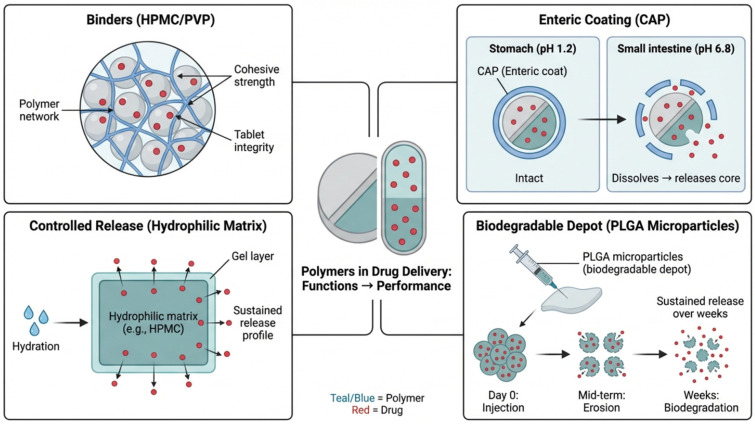
Overview of polymers used in drug delivery systems, highlighting the roles of binders (HPMC/PVP), enteric coating (CAP), controlled release matrices, and biodegradable depots (PLGA microparticles). The color coding distinguishes polymers (blue/teal) from drugs (red).

In solid dosage forms, polymers like Hydroxypropyl Methylcellulose (HPMC) and Povidone (PVP) act as binders, providing the cohesive strength necessary to maintain tablet integrity. Beyond mere “fillers,” these excipients influence the disintegration time and flowability of the powder mass [7]. The image below visually illustrates the role of these polymers, as well as other polymer types used in drug delivery systems, including coating agents and controlled release matrices. As shown in Figure 1, polymers play multiple functional roles in drug delivery systems, including improving tablet cohesion and integrity, enabling enteric protection, controlling drug release through hydrophilic matrices, and providing sustained release via biodegradable depots.

#### 3.1.2. Coating Agents

Polymeric coatings serve several purposes: protecting the drug from environmental degradation (moisture/light), masking bitter tastes, or providing enteric protection. Cellulose Acetate Phthalate (CAP) and various methacrylate polymers are commonly used materials. These polymers are insoluble in the acidic environment of the stomach but dissolve at specific pH levels in the small intestine [8].

#### 3.1.3. Controlled Release Matrices

For sustained-release profiles, drugs are embedded within a polymeric matrix. Hydrophobic polymers (like Ethylcellulose) or hydrophilic swellable polymers (like high-viscosity HPMC) control drug diffusion. As the polymer hydrates, it forms a gel layer that regulates the rate at which the active ingredient enters the systemic circulation, reducing dosing frequency and side effects [9].

#### 3.1.4. Specialized Matrices

Biodegradable polymers, such as Poly (lactic-co-glycolic acid) (PLGA), are widely utilized in injectable implants and microparticles. These matrices erode over time via hydrolysis, eliminating the need for surgical removal and providing therapeutic levels for weeks or months [10].

A summarized overview of the main polymers used in pharmaceutical manufacturing, together with their key properties and applications, is presented in Table 1.

### 3.2. Functional Properties Required

In pharmaceutical manufacturing, polymers must possess specific functional properties to ensure the safety, stability, and efficacy of the final dosage form. These properties are often categorized into physicochemical, mechanical, and biological requirements, each serving a critical role in the drug’s journey from the factory to the patient.

#### 3.2.1. Physicochemical Properties

Polymers such as methacrylate copolymers are widely engineered to exhibit pH-responsive solubility for site-specific drug delivery. The presence of functional groups, including carboxyl or amino moieties, enables these polymers to remain insoluble in the highly acidic environment of the stomach (pH ≈ 1.2) while dissolving rapidly in the neutral to slightly alkaline conditions of the small intestine (pH ≥ 6.8), thereby ensuring targeted drug release. In addition, the glass transition temperature (Tg) is a critical parameter that governs polymer flexibility and thermal behavior during processing. An appropriately selected Tg is essential for manufacturing techniques such as hot-melt extrusion (HME) and spray drying, as it ensures polymer stability and prevents thermal degradation under elevated processing temperatures [19].

#### 3.2.2. Mechanical and Processing Attributes

In tablet manufacturing, compressibility and tensile strength are critical polymer properties, as suitable polymers must exhibit good flowability and the ability to undergo plastic deformation under compression. These characteristics enable the formation of a strong and coherent tablet matrix that can withstand high-speed compression without defects such as capping or lamination. Similarly, viscosity and film-forming ability are essential parameters for coating polymers, since optimized viscosity ensures uniform atomization and consistent coating during the spraying process. An ideal coating polymer should form a continuous, flexible, and non-brittle film that provides effective protection against moisture penetration and mechanical stress, thereby enhancing the stability and durability of the final dosage form [20].

#### 3.2.3. Biological and Performance Criteria

Biocompatibility and non-toxicity are fundamental requirements for polymers used in pharmaceutical applications, as they must be pharmacologically inert, non-irritant, and non-immunogenic to ensure patient safety. In addition, controlled degradation is a critical property, particularly for injectable implants, where the hydrolysis rate of polymers such as poly (lactic-co-glycolic acid) (PLGA) must be carefully tailored to match the intended therapeutic window. Proper control of polymer degradation enables sustained and predictable drug release over the desired treatment period while minimizing adverse biological responses [21].

### 3.3. Conventional Polymer Production Challenges

While polymers are indispensable in drug delivery systems, their conventional manufacturing methods present significant challenges that compromise the reliability and safety of pharmaceutical products. These challenges mainly stem from batch-to-batch variability, scalability constraints, and environmental sustainability concerns [22].

A critical parameter that significantly influences polymer performance is the distribution of polymer chains, such as molecular weight and polydispersity index (PDI), which tells us how uniform the particle sizes are and affects how the drug is released [23]. For example, polymers with a wider molecular weight distribution or high PDI degrade faster and also have more release kinetic variations (such as initial burst release) than polymers that have a narrow molecular weight distribution, e.g., low-molecular-weight PLGA has faster degradation and a higher drug release rate [23]. Low PDI systems generally provide a more consistent and controlled drug release, whereas high PDI systems can lead to irregular and unpredictable release patterns. Review studies on PLGA-based drug delivery systems underline that intrinsic polymer properties like molecular weight and distribution are the key determinants of degradation and sustained release performance, hence the delivery system transitioning from initial release phases to prolonged release profiles [24]. On the other hand, experimental work has demonstrated that the incorporation of heterogeneity in the composition and structure of polymers can manipulate the drug release mechanisms, implying the role of polymers’ physicochemical attributes in controlling release behaviour [24].

#### 3.3.1. Batch-to-Batch Inconsistency

Traditional batch processing often experiences uneven heat and mass transfer, resulting in fluctuations in molecular weight distribution and polydispersity index (PDI). For polymers such as PLGA, even slight variations in monomer ratios can substantially alter degradation kinetics and induce an initial “burst release” of the drug, potentially leading to toxic effects [25].

#### 3.3.2. Scalability and Processing Defects

Scaling up from laboratory synthesis to metric-ton production introduces considerable mechanical and process complexities. Highly filled polymers with high drug loading frequently exhibit process-induced porosity and melt fracture during extrusion. Moreover, traditional techniques such as solvent evaporation require large quantities of organic solvents (e.g., dichloromethane), which are difficult to remove completely and may result in residual toxicity [26].

#### 3.3.3. Regulatory and Documentation Burdens

Recent compliance frameworks, including FDA guidelines, emphasize rigorous digital traceability. Nevertheless, many manufacturing facilities still rely on manual documentation, increasing the likelihood of human error. Revalidating processes to resolve these deficiencies is often prohibitively expensive, discouraging innovation and perpetuating reliance on less efficient legacy technologies.

### 3.4. Environmental and Economic Concerns

Pharmaceutical manufacturing is positioned at a critical juncture where life-saving innovation intersects with significant ecological and financial burdens. As the industry advances through 2025, balancing patient health with planetary health has shifted from a corporate social responsibility objective to an operational imperative.

#### 3.4.1. Environmental Footprint: The Hidden Toll

The pharmaceutical sector is highly resource-intensive, with carbon intensity comparable to that of the automotive and semiconductor industries. A key metric is the Environmental factor (E-factor), which quantifies the mass ratio of waste generated to the final product. For many active pharmaceutical ingredients (APIs), producing 1 kg can generate approximately 200–800 kg of waste, largely composed of hazardous solvents [27].

Moreover, environmentally persistent pharmaceutical pollutants (EPPPs), which are designed to be resilient to metabolic degradation, may also enter aquatic environments via industrial wastewater discharge. The persistence of EPPPs is a factor in antimicrobial resistance and toxicity, which are being increasingly emphasized by [28].

#### 3.4.2. Economic Pressures: The “Green” Investment Gap

Although sustainable technologies such as continuous manufacturing and biocatalysis can reduce long-term production costs by up to 15%, high initial capital expenditure remains a substantial barrier. Manufacturers therefore face a persistent economic trade-off.

Cost of procurement: The bio-based or sustainably sourced raw materials are at present estimated to be 15–25% more expensive than their petrochemical counterparts. Compliance with regulations: The increasing demands of ESG factors make it necessary for companies to invest in digital traceability solutions and sophisticated wastewater treatment, which puts a burden on generic drug manufacturers and mid-sized companies [28].

## 4. Principles of Green Synthesis

Green synthesis is one of the fundamental principles of sustainable chemistry, which has presently gained widespread attention in pharmaceutical polymer synthesis due to rising concerns of environmental, legislative, and economic pressures. The conventional method of synthesis of pharmaceutical polymers involves high energy requirements, widespread use of poisonous organic solvents, and petroleum-based raw materials. Such processes have been found to make significant contributions to pollution, health risks, and high costs of synthesis. The challenges are overcome through green synthesis, which emphasizes the optimization of reagent reduction, waste generation, and energy efficiency without compromising on the high-quality requirements of pharmaceutical polymers [29,30].

By green synthesis, one can refer to an eco-friendlier method, thereby ensuring that the chemical synthesis process is designed in a way that prevents the formation of waste, is less toxic, and more energy-efficient throughout the life cycle of the produced material. In the synthesis of pharmaceutical polymers, the design should focus on the use of less toxic raw materials, eco-friendly processing conditions, and biocompatible polymers. This is opposed to the traditional synthesis methods that were primarily concerned with yield rather than the environmental impact [31].

Twelve principles of green chemistry, first described by Anastas and Warner, provide a full set of guidelines for the application of green synthesis methods in polymer production. Even if all twelve principles play an important role in sustainable design, some of them have more significance for pharmaceutical polymers. These principles include those related to the use of renewable feedstocks, safer solvents, energy efficiency, catalysis, and design for degradation. A full set of twelve principles, along with their relevance to pharmaceutical polymers, is presented in Table 2.

Renewable resources have a key role in green polymer synthesis, and in the pharmaceutical sector, in particular, the importance of safety and biocompatibility cannot be overstated. Natural origin polymers, including starch, cellulose, chitosan, and alginate, have gained importance due to their biodegradability, non-toxicity, and acceptable regulatory status compared to traditional petroleum origin polymers. In contrast to the latter, renewable origin polymers decrease the dependence on non-renewable resources and have a lower carbon footprint. Their use in the pharmaceutical sector promotes the concept of a circular economy due to their biodegradable nature, thus preventing accumulation in the environment [36,37].

Energy efficiency is one of the most important aspects of green synthesis. The traditional method of synthesis involves high temperatures and pressures. The energy required in such synthesis is high. However, green synthesis promotes other techniques such as enzyme-catalyzed synthesis or microwave synthesis. Such techniques are carried out in less harsh conditions. The techniques are energy efficient. In addition to this, they are important in terms of control during pharmaceutical synthesis [38].

The replacement of hazardous solvents is one of the main goals of green polymer synthesis. Traditional polymer synthesis relies on volatile organic solvents that are hazardous to the environment and human health. Green synthesis focuses on the use of safer alternatives to solvents, which include water-based processes, solvent-free melt polymerization, and supercritical carbon dioxide. These methods make polymer synthesis safer by avoiding the toxicity of solvents and ensuring compliance with pharmaceutical standards related to the use of residual solvents [36,39,40].

Catalysis increases the sustainability of polymer synthesis processes through improved efficiency and selectivity, and through lower waste production. Enzyme and metal-free catalysts are more useful in pharmaceutical applications because of their high specificity and low toxicity. In addition, green synthesis enables the development of biodegradable polymers that can easily degrade into non-toxic substances after use, thereby preventing persistence and facilitating the development of safe drug delivery systems. Generally, the application of green synthesis principles offers observable economic and sustainability advantages over the conventional polymer synthesis process [41,42,43,44]. 

## 5. Green Polymer Synthesis Techniques

The change in the way drugs are made to be more environmentally friendly has fundamentally redefined the way polymers are made. The functional performance of the polymer is no longer the only thing that matters. Now, the material’s “cradle-to-grave” environmental footprint is furthermore an important design factor. This part talks about the change from traditional polymerization methods that use oil to new, eco-friendly ones that use less energy and are less harmful [45].

### 5.1. Biopolymers (Chitosan, Alginate, Starch-Based Polymers)

**Figure 2:**
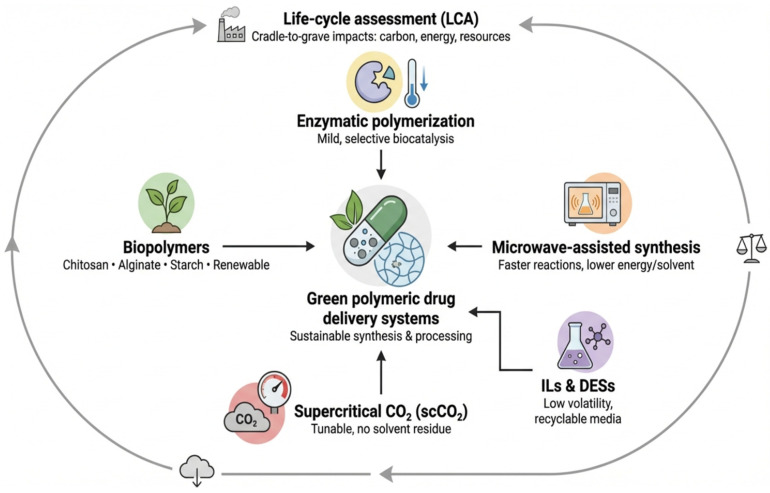
Overview of green polymer synthesis techniques. Sustainable polymeric drug delivery systems are driven by renewable biopolymers, mild biocatalysis, energy-efficient methods, and green solvents, all continuously evaluated through a cradle-to-grave Life-Cycle Assessment (LCA).

The creation of biopolymers like chitosan, alginate, and starch-based polymers is the basis for making polymers in a way that is good for the environment in the pharmaceutical field. These polymers do not come from petrochemicals; instead, they come from renewable biological waste products like plant starches, brown seaweed (alginate), and crustacean shells (chitin → chitosan). Chitosan is a good choice for drug delivery systems and tissue scaffolds because it is highly biocompatible, biodegradable, and bioactive. Also, it can be changed chemically to get certain functional properties [46]. Alginate is a natural polymer that comes from brown seaweed. When it meets calcium ions, it forms three-dimensional gels that can be used to deliver drugs in a controlled way. It does not need dangerous organic solvents to be processed. Starch-based polymers are a cheap and biodegradable option that can be changed through mild chemical reactions to get the right mechanical and thermal properties [34]. Recent research (2021–2025) has concentrated on employing green processing techniques to enhance the characteristics of these polymers, including the partial substitution of enzymes for aggressive alkali treatments in chitosan extraction, thereby minimizing energy consumption and chemical waste, one of the principal applications of green chemistry principles [47]. As illustrated in Figure 2, green polymeric drug delivery systems integrate renewable biopolymers, enzymatic polymerization, microwave-assisted synthesis, ionic liquids/deep eutectic solvents, and supercritical CO_2_ within a sustainability-oriented framework that can be evaluated through life-cycle assessment.

### 5.2. Enzymatic Polymerization

Enzymatic polymerization is a big change in how polymers are made because it lets polymer chains form in mild conditions (low temperature and pressure, water, and no toxic solvents). Enzymes are biocatalysts that do not use metal catalysts or organic solvents. This is in line with the principles of sustainability and reducing waste [48]. Enzymatic polymerization is used in the pharmaceutical industry to make functional polymers that improve drug delivery systems by controlling how quickly they dissolve and release. For instance, amino acids or sugar derivatives can be linked together to make polymers that break down in the body. One of the best things about this method is that it makes polymer chains very selective. This cuts down on uncontrolled polymerization and the need for extra, costly steps to clean up the environment [49].

### 5.3. Microwave-Assisted Synthesis

Microwave-assisted polymer synthesis is a good way to make green polymers because it speeds up reactions and uses less energy than other methods. Studies have shown that biopolymers like sodium alginate-chitosan gels can be made in just one step using only microwaves. This cuts down on the need for dangerous solvents and speeds up the reaction time and energy use [50]. When you hear something in a microwave, the rapid and uniform volumetric heating throughout the reactive medium. This speeds up the process of forming molecular networks between polymer chains. This method not only makes production more efficient, but it also helps design drug delivery systems that are easier to break down and better at controlling how drugs are released [51].

### 5.4. Supercritical CO_2_ Techniques

Supercritical carbon dioxide (scCO_2_) is an advanced green processing option because it can dissolve and shape polymers without using traditional organic solvents, which are often dangerous and flammable. When CO_2_ is in a supercritical state, it has properties that are between those of gases and liquids. This means that it can be very permeable and have different levels of solubility, which can be used to change the structure of polymers [52]. You can use supercritical CO_2_ techniques to make polymer films, gels, or nanoparticles that are useful in medicine. The process has a smaller carbon footprint, less hazardous waste, and the ability to recycle CO_2_ within the system [53].

### 5.5. Ionic Liquids & Deep Eutectic Solvents

Polymer manufacturing has a big impact on the environment because of the solvents that are used. Replacing them with ionic liquids (ILs) or deep eutectic solvents (DESs) is better for the environment because these solvents are usually not flammable, have low vapor pressure, and can be used again [53]. In recent years, there has been a growing interest in using natural DESs to get polymers like chitosan and chitin out of crustacean waste. These methods have become more efficient while using fewer harmful chemicals. These solvents also help make green polymers by providing an environmentally friendly way to polymerize or change polymer chains. For example, DESs are used as solvents to make nanopolymeric materials. They help control solubility, viscosity, and chemical reactivity without making any toxic waste [54]. To strengthen the discussion of microwave-assisted synthesis and ionic liquid/deep eutectic solvent processing, representative quantitative comparisons are summarized in Table 3. According to recent studies, microwave-assisted methods consistently shorten processing time and may improve yield or product quality, whereas DES/NADES-based systems frequently enhance polymer purity and reduce dependence on harsh reagents. However, direct comparison across studies remains challenging because outcomes depend strongly on feedstock type, solvent composition, microwave power, and purification workflow [55].

### 5.6. Life-Cycle Assessment (LCA) of Green Polymer Processes

Life-cycle assessment (LCA) has become an important way to learn about the environmental and economic effects of making green polymers from start to finish. LCA looks at how much energy is used, how much carbon is released, how resources are used, and how waste is handled at every step of the value chain [60]. LCA helps the pharmaceutical industry choose methods that have less impact on the environment without lowering performance or safety. When you compare traditional chitosan extraction methods with greener enzyme- or solvent-based methods; for example, you can see that using greener methods produces a lot less solvent waste and carbon emissions [61]. Relying on LCA also pushes researchers and manufacturers to use production designs that use less energy and resources. This sets standards that can be used to judge how sustainable polymers are from both an industrial and a pharmaceutical point of view [62]. A comparative overview of conventional and green polymers is presented in Table 4, emphasizing key differences in carbon emissions, energy consumption, and environmental impact. As shown, green polymer production strategies generally reduce CO_2_ emissions and energy demand while minimizing long-term environmental persistence, thereby supporting more sustainable pharmaceutical manufacturing systems.

## 6. Sustainability in Polymer Manufacturing

You need to think about the whole life cycle if you want to make pharmaceutical polymers in a way that is good for the environment. To have the least effect on the environment, manufacturers are focusing on green chemistry methods and biodegradable, renewable polymers [65]. Biopolymers made from cellulose, starch, and chitosan are one example. These are all things that come from farming or forestry. They are used as multifunctional excipients that reduce the need for fossil feedstocks [66]. Most of the time, these bio-based polymers break down safely into water, CO_2_, and biomass after they are used. This makes it easier to throw away things you do not need anymore [67].

**Figure 3:**
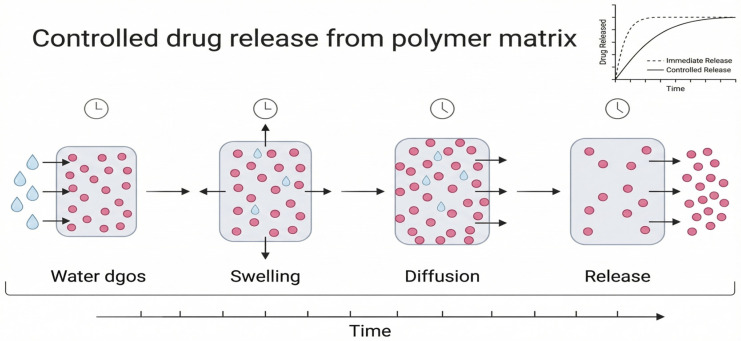
Comparison between conventional and green polymer production in pharmaceutical manufacturing. Conventional methods depend on petrochemicals, high energy, and toxic solvents, leading to high waste and emissions. In contrast, green approaches use renewable resources, energy-efficient techniques, and eco-friendly solvents to reduce environmental impact.

Also, processing and additives are improved: solvent use is kept to a minimum, and wherever possible, dangerous reagents are replaced with water- or enzyme-based methods [68]. Life-cycle analyses consistently reveal that electricity and solvent consumption constitute the predominant components of environmental footprints; thus, the transition to renewable energy and environmentally benign chemicals is imperative [69]. To make pharmaceutical polymers sustainable, you need to combine renewable materials with manufacturing methods that use less energy and produce less waste [70]. The transition from conventional polymer production to sustainable green approaches is illustrated in Figure 3.

### 6.1. Environmental Sustainability Metrics

Environmental sustainability in polymer production is assessed through carbon footprint reduction, waste minimization, and circularity. Carbon emissions remain a major concern because solvent use and heating account for much of the CO_2_ generated during polymer processing [71]. Life-cycle studies have shown that the production of APIs and excipients generates an average of 34 kg CO_2_-eq per unit, mainly from chemical inputs and energy use [69]. Pharmaceutical manufacturing emissions were also reported to be 55% higher than those of the automotive sector [72]. In this context, bio-based polymers may help lower net emissions by storing carbon captured during biomass growth [73]. Broader sustainability strategies, including renewable energy, recycling, and process innovation, have been reported to reduce carbon footprints by up to 30% [74]. with these improvements increasingly monitored through carbon footprint reporting systems [75].

Waste minimization is another key metric. Process intensification, continuous manufacturing, solvent recycling, and in-line monitoring can reduce solvent losses, byproducts, and overall waste generation [76]. Case studies have shown that combining green chemistry with recycling systems can reduce process waste by up to 50% and energy use by 40% [77]. However, some solutions involve trade-offs; for example, single-use bioreactors reduce water and cleaning waste but increase plastic consumables [78], although they may still use less water and energy than steel systems [79].

Circularity further strengthens sustainability by promoting reuse, recycling, and upcycling instead of disposal. Advanced recycling methods now allow PET waste to be broken down and reprocessed with conversion efficiencies above 99% [80,81]. while post-consumer plastics are increasingly reused as polymer feedstocks [82]. In pharmaceutical packaging, take-back and recycling systems have been associated with lower life-cycle impacts than landfilling [83]. In addition, lightweighting and eliminating unnecessary material layers can reduce energy demand and emissions during polymer production [84].

### 6.2. Circular Economy Concepts

Recent research has shown specific examples of how to “upcycle” raw materials into pharmaceutical polymers. For example, agricultural waste has been turned into useful polymer adhesives for transdermal drug patches [85]. We chemically process crop waste into a polymer that can be used instead of a synthetic binder to make this agro-waste adhesive (shown above). These bio-based excipients show how biomass is not food that can be used in the pharmaceutical value chain. Many traditional polymer excipients, such as vinylpyrrolidone or Eudragit copolymers, exhibit negligible biodegradation, indicating their persistence upon disposal. This shows why recycling or reusing is so important: polymers should be saved instead of burned. In that sense, progress in general polymer recycling is a source of inspiration [86]. Engineers are looking into closed-loop solvent and polymer recovery systems on-site in the pharmaceutical industry. Even though there isn’t much infrastructure for recycling excipients yet, these efforts, along with making new excipients from recyclable feedstocks, are moving us closer to a more circular outcome [87].

### 6.3. Recycling and Upcycling of Polymeric Excipients

**Figure 4:**
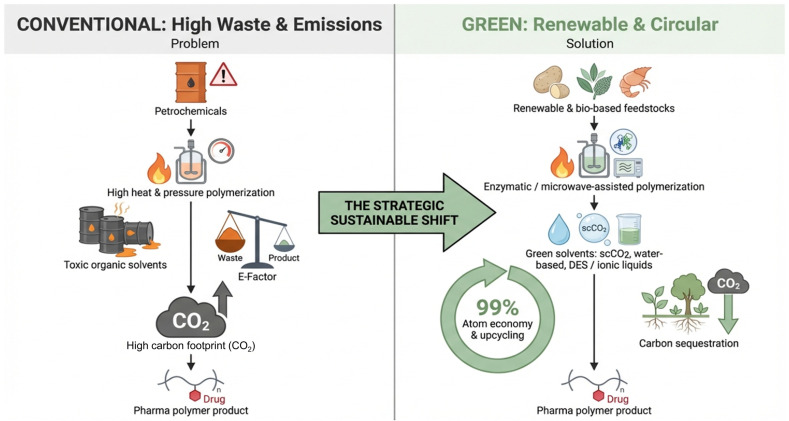
Comparison between conventional polymer synthesis methods (**left**) and green, renewable, and circular solutions (**right**). The image highlights the transition to more sustainable manufacturing approaches, focusing on renewable feedstocks, green solvents, and energy-efficient processes.

But the green transition is taking longer because it is hard to put the plan into action. Because of strict quality and regulatory controls, it is hard for the pharmaceutical industry to change materials or processes on the fly. It takes more time and money to make sure that a new polymer grade is safe and that recycled materials meet the pharmacopeia’s standards for purity [88]. Experts also say that the rules in the field are not clear. There is not a single certification for “eco-friendly” excipients, and claims about sustainable polymers are not always clear. There are not enough good recyclers for packaging with more than one layer, which is another problem. Most recycling systems cannot separate out complicated pharmaceutical films or tools that can only be used once [89]. Overcoming these hurdles will require coordinated action: collaboration among drugmakers, regulators, and recyclers is needed to establish a clear “sustainability by design” framework. Efforts like the EU ENKORE project (developing eco-design guidelines and digital material passports) exemplify how multi-stakeholder innovation can set new standards [90]. As shown in Figure 4, green polymer synthesis offers a sustainable alternative to conventional routes by reducing waste generation, lowering carbon emissions, improving atom economy, and promoting the use of renewable bio-based resources. It is important to distinguish between recycling strategies for packaging polymers and those applicable to active polymeric systems used in pharmaceutical formulations or delivery platforms. Packaging polymers primarily serve a protective and containment role and are therefore evaluated within container–closure, packaging compatibility, stability, and waste-management frameworks [91]. In contrast, active polymer systems such as excipients, drug-release matrices, hydrogels, nanoparticles, and implantable carriers directly influence drug performance, patient exposure, and product safety. As a result, regulatory expectations for active polymer systems are substantially stricter, extending beyond material recycling to include GMP compliance, quality and purity control, extractables and leachables assessment, biocompatibility, stability, and, where relevant, clinical performance. From a circularity perspective, packaging polymers may enter reuse or recycling streams if material integrity and safety are maintained, whereas active polymer systems generally cannot be treated as ordinary recyclable materials because any structural or chemical variability may alter therapeutic behavior. Thus, although both categories fall under the broader sustainability discussion, they differ fundamentally in function, risk profile, and regulatory burden [92]. Accordingly, sustainability frameworks in pharmaceutical manufacturing should avoid treating packaging polymers and active polymer systems as a single recycling category, because each is governed by distinct technical and regulatory requirements [91].

## 7. Applications in Pharmaceutical Manufacturing

Polymers are very important for making drugs today because they can be used in many different ways to make drugs, build drugs, and deliver drugs. They have led to new ways to give drugs, make things more stable, and get patients to follow the rules because they can be made for certain tasks. In the last ten years, especially since 2020, research has been focused on both performance and sustainability. This means using polymer technologies that are better for the environment, break down naturally, and are safe for living things [93].

### 7.1. Controlled Drug Release Systems

Polymers are very useful in medicine because they help control how drugs are released. Most dosage forms release active ingredients quickly, which can change the level of plasma and make you need to take the medicine more often. On the other hand, controlled release systems that use polymers give drugs in a steady or targeted way. This keeps the therapeutic levels high and lowers the chance of side effects. These systems often use polymers that break down when they come into contact with water or enzymes. The drug can be slowly released as the polymer matrix breaks down. Researchers are testing different materials to see if they can be used to give drugs in a controlled or zero-order way. Polylactic acid (PLA), poly (lactic-co-glycolic acid) (PLGA), and polyanhydrides are some examples [82,94].

Natural and synthetic polymers can change how things are released, such as by breaking down, swelling, or diffusing. Researchers have made new polymeric matrices that react to changes in the body’s chemistry, such as changes in pH. This makes sure that bioavailability meets clinical needs. This changes how chronic diseases, cancer, and localized therapies are treated [95].

### 7.2. Coating Materials

Polymer coatings do two things for pharmaceutical products. They protect the drug from things like moisture, light, and oxidation in the environment. Second, they allow for controlled disintegration, which protects the active ingredient in the stomach and releases it in the intestine, or the other way around [96].

Recent research in sustainable polymer coatings focuses on bio-based and biodegradable materials to replace conventional petroleum-derived polymers. These renewable coatings are engineered to maintain functional performance while reducing environmental impact and improving end-of-life degradation [97].

In pharmaceutical tablets, coatings made from cellulose derivatives or other bio-based polymers not only provide protective barriers but also contribute to altered drug release profiles (enteric or sustained release). Advances also include antimicrobial and functional coatings that improve safety during storage and handling [8].

### 7.3. Hydrogels

Hydrogels are three-dimensional polymer networks capable of absorbing significant amounts of water. They mimic biological tissues and are particularly useful when water content, flexibility, and biocompatibility are essential. In drug delivery, hydrogels can be loaded with therapeutic agents and designed to release them over time through controlled swelling and diffusion [98]. Natural polymer-based hydrogels such as those derived from chitosan, alginate, and hyaluronic acid offer advantages in biodegradability and non-toxicity, making them ideal for implantation or topical administration [99]. Their swelling properties can be tuned by altering crosslink density, enabling precise control over release kinetics to meet therapeutic needs. Hydrogels also find applications beyond drug release: as scaffolds for tissue engineering, biosensors, and wound healing matrices due to their similarity to natural extracellular matrices and their ability to support cell growth [98].

### 7.4. Nano Polymers

**Figure 5:**
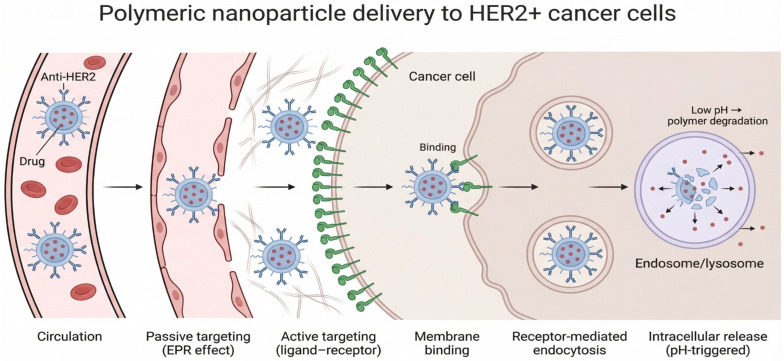
Schematic illustration of polymeric nanoparticle-based targeted drug delivery to HER2-positive cancer cells. The diagram demonstrates systemic circulation, passive targeting via the enhanced permeability and retention (EPR) effect, active ligand–receptor targeting (Anti-HER2), cellular binding, receptor-mediated endocytosis, and pH-triggered intracellular drug release within endosomes/lysosomes.

Nanotechnology has expanded the utility of polymers into nanoparticulate drug carriers. Polymeric nanoparticles enhance drug solubility, protect labile drugs, improve distribution, and facilitate targeted delivery to specific tissues or cells [100]. Biodegradable nano polymers, such as those based on PLA, PLGA, and polysaccharides, are engineered to deliver proteins, peptides, vaccines, and small molecules with enhanced stability and reduced systemic toxicity. These nano-systems can be further functionalized with ligands or antibodies to achieve targeted drug delivery, a key strategy in cancer therapy and personalized medicine [100]. The integration of polymer science with nanotechnology has opened pathways for personalized drug delivery platforms, as shown in Figure 5 [101].

### 7.5. Polymer Modification for Advanced Medical Applications

Polymer modification has become a key strategy for expanding the medical utility of both natural and synthetic polymers. By introducing functional groups or blending polymers with bioactive moieties, researchers can tailor antimicrobial activity, mucoadhesion, degradability, drug-loading efficiency, and stimulus responsiveness. In antimicrobial applications, modified polymers can disrupt microbial membranes, reduce biofilm formation, and improve the safety of wound dressings, coatings, and implantable systems [102]. In drug delivery, chemical modification enables more precise control over release kinetics, site-specific targeting, and improved interaction with biological barriers. Representative examples include thiolated, catechol-, methacrylate-, and maleimide-functionalized chitosan derivatives, which show enhanced mucoadhesion and transmucosal transport, as well as polymer-functionalized mesoporous systems that provide pH-, redox-, enzyme-, or temperature-responsive release. Therefore, polymer modification should be considered not merely a structural adjustment, but a translational design tool that links polymer chemistry with therapeutic performance, safety, and application-specific functionality [103].

## 8. Pharmacoeconomics of Green Polymers

### 8.1. Cost–Benefit Analysis

The incorporation of green polymers in the pharmaceutical industry requires a thorough cost–benefit analysis to support the shift in the paradigm from the conventional petroleum-based materials. First, the use of green materials requires a high capital outlay for research and development, testing of the materials, and the modification of the existing production lines [104]. However, the long-term benefits of the use of green materials far outweigh the initial costs. The use of green polymers like PLA and PHA reduces the costs of waste management and the financial risks associated with environmental compliance [105]. In addition, the proactive approach towards the use of green materials improves the CSR image of organizations, which may lead to an increase in the market share of environmentally conscious consumers [106]. From a macroeconomic perspective, the cost–benefit analysis is gradually shifting in favor of biopolymers as the economies of scale are achieved and the financial costs of carbon emissions from conventional plastics rise through global taxation. In addition to the costs, the intangible benefits are in favor of the green revolution.

### 8.2. Manufacturing Cost Comparison (Green vs. Conventional)

When comparing the direct manufacturing cost of green polymers with traditional synthetic polymers, there is a considerable price difference in the market at present. Traditional petroleum-based plastics enjoy the benefits of highly optimized, heavily subsidized, and industrialized manufacturing processes that have been in place for several decades, making them extremely cheap to buy [107]. On the other hand, advanced biopolymers tend to have a higher price range, which is considerably higher than conventional plastics due to complex, non-industrialized polymerization processes and the unavailability of raw materials [108]. However, a cost comparison needs to be done over the entire manufacturing process. Traditional manufacturing processes tend to involve enormous amounts of toxic organic solvents that are expensive to buy and even more expensive to dispose of properly, as per strict regulations [109]. On the other hand, green polymers tend to involve aqueous processing or solvent-free melt compounding based on green chemistry, which does not require the use of expensive hazardous materials, thus bridging the cost difference during the active formulation process [110]. Ultimately, although the cost of acquiring raw materials for green polymers is still higher, the operational cost savings in waste disposal, energy efficiency, and reduced safety measures offer a highly competitive total cost of manufacturing scenario [111].

### 8.3. Long-Term Economic Benefits

The overall long-term economic benefits of the shift to green polymers in pharmaceutical production are very significant and far-reaching. As the global regulatory environment continues to impose harsher penalties on carbon-intensive business models, pharmaceutical companies that adopt bio-based polymers will automatically stay clear of high carbon taxes and future regulatory compliance costs [112]. In addition, governments around the world are actively providing tax breaks, research funding, and production subsidies to pharmaceutical companies that are proactive in adopting sustainable and circular economy business models, thereby enhancing long-term net profitability [113]. In addition to financial regulatory benefits, green polymers have been shown to have high biocompatibility, which can significantly shorten the stringent clinical trial process for new drug delivery systems by minimizing immune system reactions, thereby accelerating commercialization timelines [114]. This accelerated commercialization directly leads to extended patent life and uninterrupted revenue streams for blockbuster pharmaceuticals. Looking ahead over a decadal timeframe, the price stability of renewable organic materials compared to the price volatility of crude oil guarantees a much more stable economic forecasting platform for large pharmaceutical companies [115].

### 8.4. Impact on Supply Chain

The integration of green polymers is a significant disruptor, but ultimately an enhancer, of the existing pharmaceutical supply chain’s resilience. The traditional supply chain is strongly linked to the petrochemical industry, rendering it extremely susceptible to geopolitical tensions and extremely fluctuating prices of fossil fuels [106]. With the advent of biopolymers from agricultural by-products, sustainable cellulose, or microbial fermentation, pharmaceutical companies can proactively work towards diversifying their supply chains and localizing them. This localization effectively counters the monetary risks of global shipping disruptions and significantly reduces the carbon footprint associated with long-distance transportation [115]. However, the transition phase itself poses logistical supply chain issues, primarily the pressing need to develop effective procurement infrastructure for the regular sourcing of high-quality, standardized biological materials [113]. In addition, novel storage and cold chain handling practices need to be implemented, as certain biodegradable polymers have varying thermal resistance and moisture content characteristics compared to their more resilient synthetic counterparts [116].

### 8.5. Investment Barriers

Notwithstanding the highly evident environmental benefits, a number of daunting investment hurdles currently exist to impede the widespread and rapid acceptance of green polymers within the pharmaceutical industry. The first and foremost challenge that still persists is the enormous initial capital investment required to modify the existing production infrastructure [104]. The existing extrusion, molding, and packaging machinery is designed exclusively for conventional plastics; modifying these multi-million-dollar machines to accommodate the particular melt viscosities of bio-nanocomposites is a technological challenge that demands significant investment [105]. The second major investment hurdle is the extensive R&D investment required to ensure that these new materials comply with the extremely stringent safety, sterility, and storage requirements set by the regulatory bodies [117]. Venture capitalists and in-house investors are often unwilling to invest in such transitions due to the risk of rejection or delayed approval by the regulatory bodies. Additionally, the absence of large-scale industrialization of many biopolymers currently in development creates a physically unstable supply chain, causing conservative pharmaceutical industry leaders to hold back on full-scale adoption without guaranteed access to the materials.

### 8.6. Health–Economic Evaluation (Cost per QALY, Cost Savings, etc.)

The full health–economic analysis of green polymers would involve the cost analysis extending far beyond the production process to actual patient outcomes and societal healthcare costs. The use of advanced biopolymers in targeted drug delivery systems and theranostic nanoformulations would greatly improve the efficacy of drugs while ensuring minimal systemic off-target toxicity [112]. By greatly improving patient compliance and reducing the rate of adverse drug reactions, these green materials would directly reduce the financial costs of hospital readmissions and secondary treatments [114]. As such, the use of green polymers in healthcare would ensure a highly favorable Incremental Cost-Effectiveness Ratio (ICER) and reduce the cost per Quality-Adjusted Life Year (QALY) gained, thoroughly justifying the higher initial costs of these materials [115]. Furthermore, the public health benefits of reducing pharmaceutical microplastic pollution and antimicrobial resistance in global water supplies would ensure massive, albeit indirect, societal cost savings [107]. Ultimately, these health–economic metrics would ensure a compelling case for the inclusion of green polymers as a vital value-based healthcare initiative rather than a simple manufacturing alternative [109].

## 9. Regulatory Perspectives on Sustainability in Pharmaceutical Manufacturing

Regulatory frameworks for sustainability in pharmaceutical manufacturing are rapidly evolving as environmental impacts and climate concerns become central to health policy [118]. Traditional regulations focused on product quality and patient safety are being expanded to incorporate environmental stewardship, waste reduction, and lifecycle responsibility [119]. For instance, in the United States, the FDA integrates sustainability into manufacturing guidance by aligning with Environmental, Social, and Governance (ESG) criteria, emphasizing green chemistry adoption and reduced environmental footprint as part of compliance strategies [120].

In the United States, the FDA’s current commitments include adopting advanced manufacturing technologies and continuously updating guidance documents to reflect environmental and sustainable priorities. For example, the FDA publishes and revises guidance for innovative manufacturing processes such as continuous manufacturing (ICH Q13), which further encourages sustainability in production and reduces waste. These advances emerge in the context of broader legislation like the Food and Drug Omnibus Reform Act, which delegates authority to support sustainable technologies and quality innovations in manufacturing plants [119].

The FDA’s approach to sustainability also extends to compliance documentation and regulatory submissions, encouraging pharmaceutical companies to demonstrate environmental responsibility in filings. This includes evaluating impacts on resource use, emissions, and waste management under environmental assessment requirements that accompany marketing and post-approval actions. Integrating these considerations into regulatory submissions strengthens the regulatory decision-making ecosystem and aligns product approval with broader sustainability goals [121].

In Europe, the European Medicines Agency (EMA) has introduced significant changes to incorporate sustainability into the pharmaceutical regulatory environment. EMA’s efforts include embedding environmental risk assessment (ERA) requirements into the approval process for medicinal products. As of 1 September 2024, an updated EMA guideline mandates that all marketing authorization applications include detailed environmental risk evaluations to protect ecosystems from pharmaceutical residues and mitigate long-term environmental harm [122].

EMA’s environmental risk guideline outlines a stepwise evaluation of environmental exposure, fate, and ecotoxicological impact of medicinal product constituents. These assessments are legally required for all new human medicines, including generic products, reinforcing environmental stewardship as a regulatory obligation rather than voluntary best practice [123].

Beyond ERA, the EMA’s broader regulatory reform agenda explicitly prioritizes sustainability. In late 2025, the European Parliament and Council reached a political agreement on new EU pharmaceutical legislation that will modernize regulatory frameworks to support innovation while enforcing environmental sustainability metrics across the lifecycle of medicines from development through post-market surveillance [124].

The EMA’s regulatory stance continues to emphasize the importance of sustainable technologies such as continuous manufacturing and green chemistry to meet both quality and environmental objectives. By harmonizing sustainability expectations with Good Manufacturing Practices (GMP) standards, EMA promotes resource efficiency, waste minimization, and compliance with environmental safety thresholds within the EU pharmaceutical sector [124].

At the international harmonization level, the International Council for Harmonisation (ICH) plays a pivotal role in shaping regulatory expectations that indirectly support sustainability goals. Although ICH guidelines like Q8–Q11 primarily address quality and lifecycle management, their science- and risk-based frameworks promote efficient manufacturing processes, reduced material waste, and lifecycle thinking, which dovetail with environmental objectives. For example, ICH Q13’s focus on continuous manufacturing encourages reductions in solvent use, energy consumption, and overall environmental impact [120].

Regulatory dialogue also extends to operational frameworks such as Quality by Design (QbD) and Quality by Digital Design (QbDD), which help embed sustainability into regulatory compliance workflows. These integrated approaches permit regulators like the FDA and EMA to assess sustainability metrics alongside traditional quality and safety factors, thereby facilitating regulatory approval pathways that reward environmentally responsible manufacturing choices [121].

In addition to formal regulatory bodies, global health organizations influence sustainability expectations. For example, the World Health Organization (WHO) has issued calls for transformative action toward greener pharmaceutical production, advocating for regulatory standards that emphasize environmental impact reductions and digital approaches to strengthen sustainable manufacturing practices worldwide [121].

Regulatory perspectives increasingly emphasize supply chain resilience and ESG integration. Regulatory Affairs professionals are now expected to incorporate environmental goals into CMC (Chemistry, Manufacturing, and Controls) strategies and submissions, aligning corporate sustainability reporting with regulatory expectations. This trend reflects the growing regulatory frontier where sustainability metrics influence decision-making and product lifecycle strategies [121].

Despite these advances, challenges remain. Regulatory harmonization across regions is ongoing, with differences in sustainability requirements and enforcement mechanisms between the FDA, EMA, and other global health authorities. Harmonized frameworks could reduce the complexity of global compliance and foster greater uptake of sustainable manufacturing practices across markets [3].

In summary, regulatory perspectives on sustainability in pharmaceutical manufacturing have evolved from a peripheral consideration to a central factor in approval and compliance strategies. The FDA, EMA, and ICH are progressively incorporating environmental, lifecycle, and ESG considerations into regulatory guidance, environmental risk requirements, and quality frameworks. As regulatory expectations align with global sustainability goals, pharmaceutical companies are increasingly accountable for demonstrating environmental responsibility alongside product safety and efficacy in regulatory submissions [122].

## 10. Challenges and Limitations

Despite the notable environmental and safety benefits of green polymer production, various hurdles hinder its broader implementation in pharmaceutical manufacturing. A thorough assessment of these challenges is necessary within a review framework to offer a fair and realistic evaluation of the domain. One major obstacle is the complexity involved in scaling up green synthesis methods. Although numerous green polymerization strategies demonstrate impressive efficiency and selectivity in laboratory settings, adapting these processes for industrial-scale production often encounters challenges related to reproducibility, process consistency, and precise reaction management. Advanced methods like enzymatic and microwave-assisted polymerization typically demand specialized machinery and intricate monitoring systems, which increases the complexity of the scale-up process [123].

The price of sustainable raw materials poses another significant challenge. Renewable and bio-based resources are frequently pricier than traditional petroleum-based materials, and their availability can be influenced by fluctuations in agricultural productivity and constraints within the supply chain. These elements can increase production expenses and reduce the economic viability of green polymers, especially in large-scale pharmaceutical production, where cost-effectiveness is crucial. While long-term savings might be realized through lower waste management and compliance costs with regulations, the initial financial impact continues to be a considerable obstacle [123].

The uptake of green technologies in the industrial sector is additionally hindered by the lack of change acceptance and the necessary infrastructure. In the pharmaceutical sector, which is highly regulated, there is often reluctance to adopt changes in existing technologies due to concerns about consistency, regulatory compliance, and return on investment. The need to validate the entire process may slow the rate of adopting green synthesis technologies, especially when compared to well-established traditional technologies.

Performance-related constraints also limit the use of green polymers. Some bio-based and biodegradable polymers show lower stability in terms of their chemical, thermal, or mechanical properties when compared to traditional synthetic polymers. This can affect shelf life, drug release properties, and performance. It remains a major challenge to ensure that green polymers meet strict pharmaceutical standards in terms of their performance and quality [36].

Another set of factors that add to the hindrances is those related to regulations and validation. The approval process for new green polymeric excipients requires information about their safety, toxicity, and stability, even long-term studies. The fact that there is little available long-term information for most new green polymers might make them resistant to approval by organizations like the U.S. Food and Drug Administration (FDA) or the European Medicines Agency (EMA) [124].

Furthermore, the minimal economic motivation will reduce the incentive to develop green polymers. The lack of government support, tax incentives, and enforcement of policies will impede the shift to sustainable practices. Without stronger economic and regulatory forces, the use of green polymers will not be fully adopted, despite the significant environmentally beneficial potential [124].

## 11. Conclusions

The need for sustainable green polymer production in the pharmaceutical industry is imperative for environmental and economic sustainability. Polymers are essential materials in the pharmaceutical industry for drug delivery systems, packaging, and medical devices. However, the traditional use of petrochemical-based polymers has raised major concerns because of their environmental toxicity, high energy consumption, and persistence in the environment after disposal. With the increasing sustainability requirements and regulations, the pharmaceutical industry needs to look for greener alternatives that do not affect material properties.

The principles of green chemistry, which focus on the use of renewable materials, non-toxic solvents, and energy-efficient processes, provide a means of reducing the environmental effects of pharmaceutical polymers. Green chemistry practices, such as enzymatic polymerization and microwave-assisted polymerization, are also more environmentally friendly and energy-efficient compared to traditional methods. Green chemistry practices and principles provide a means of reducing the environmental effects of pharmaceutical polymers. For example, bio-based and biodegradable polymers, which are derived from renewable resources such as chitosan, alginate, and starch, are more environmentally friendly compared to their petroleum-based counterparts because they are non-toxic, biodegradable, and have a lower carbon footprint.

Life cycle assessment (LCA) is a critical tool in the evaluation of the environmental performance of green polymer technology. LCA is a process that evaluates the environmental effects of a polymer from the raw material stage to the disposal stage. For example, biopolymers such as PLA (polylactic acid) and PHAs (polyhydroxyalkanoates) have demonstrated a substantial reduction in carbon emissions and energy consumption compared to traditional plastics. The use of LCA in decision-making provides a means for pharmaceutical companies to choose materials and processes that are environmentally friendly while still meeting the required standards.

Apart from the environmental advantages, cost–benefit analysis and techno-economic studies are essential for identifying the viability of implementing green polymers on a larger scale. Although the initial investment costs for green polymer technology are higher, particularly in terms of material and research and development expenditure, the long-term economic advantages can be significant. The decrease in waste management costs, energy savings, and adherence to stringent government regulations can help counterbalance the higher initial investment. Moreover, green polymers can also serve as a differentiating factor for companies to target eco-friendly consumers and improve their corporate social responsibility (CSR) image.

However, some challenges are currently impeding the large-scale implementation of green polymers in the pharmaceutical industry. Scalability is a major issue, as most green polymerization techniques, including enzymatic polymerization and microwave-assisted polymerization, are struggling to be adapted from laboratory to industrial scales. These techniques also demand sophisticated equipment and monitoring systems, making them more complex and expensive on an industrial scale. In addition, the increased cost of sustainable raw materials, as well as uncertainties in agricultural productivity, are other economic constraints. Although biodegradable polymers from renewable resources have long-term economic and environmental advantages, the initial investment cost is a major hindrance.

Performance-related issues are also a hindrance to the use of green polymers. Some bio-based polymers have shown reduced stability in terms of their chemical, thermal, or mechanical properties compared to traditional synthetic polymers. It is essential to ensure that these polymers meet the stringent pharmaceutical requirements for drug release kinetics, shelf life, and mechanical strength. Furthermore, the absence of long-term safety and biodegradability information for most green polymers is a regulatory barrier, as the FDA and EMA demand a vast amount of information before approving new materials for pharmaceutical applications.

To overcome these issues, there is a need for cooperation between all stakeholders, including suppliers, manufacturers, and regulatory bodies. Governments can help facilitate the shift by offering financial incentives, tax breaks, and subsidies to companies adopting green technology. In addition, the need for investment in research and development to enhance the scalability, performance, and safety aspects of green polymers cannot be overemphasized. Advances in manufacturing processes, including continuous manufacturing, solvent recovery, and recycling of polymers, will further help minimize waste and energy consumption, thus ensuring a sustainable pharmaceutical supply chain.

## Figures and Tables

**Table 1:** Summary of main polymers used in pharmaceutical manufacturing, their properties and applications.

Polymer	Key Properties	Main Applications	References
HPMC	Hydrophilic, gel-forming, biocompatible, good film-forming ability	Binder, controlled-release matrix, coating	[11]
PVP	Water-soluble, inert, adhesive, good stabilizing/solubilizing ability	Binder, solubility enhancer, stabilizer	[12]
Methacrylate copolymers (Eudragit^®^)	pH-responsive, film-forming, tunable release behavior	Enteric coating, site-specific and controlled release	[13]
PLGA	Biocompatible, biodegradable, controllable degradation and release	Microparticles, nanoparticles, implants, injectable depots	[14]
PLA	Biodegradable, biocompatible, suitable mechanical strength	Drug carriers, implants, nano-delivery systems	[15]
PHA	Bio-based, biodegradable, biocompatible, useful for sustained delivery	Nanocarriers, drug delivery, biomedical systems	[16]
Chitosan	Biodegradable, biocompatible, mucoadhesive, bioactive	Oral drug delivery, hydrogels, scaffolds, nanoparticles	[17]
Alginate	Gel-forming, biocompatible, mild processing, swelling behavior	Encapsulation, matrix tablets, hydrogels, controlled release	[18]

**Table 2:** The Twelve Principles of Green Chemistry and Their Relevance to Pharmaceutical Polymer Manufacturing.

Principle	Description	Relevance to Pharmaceutical Polymers	References
Prevention	Prevent waste rather than treat it	Reduction of polymerization by-products	[4]
Atom economy	Maximize incorporation of materials	High-yield polymer	[4]
Less hazardous synthesis	Use substances with low toxicity	Safer polymer production routes	[4]
Design safer chemicals	Maintain function with minimal toxicity	Water-based and solvent-free systems	[4]
Safer solvents	Avoid hazardous solvents	Water-based and solvent-free systems	[4]
Energy efficiency	Minimize energy requirements	Low-temperature polymerization	[4]
Renewable feedstocks	Use renewable raw materials	Starch-, cellulose-, chitosan-based polymers	[32]
Reduce derivatives	Avoid unnecessary steps	Simplified polymer synthesis	[4]
Catalysis	Use catalytic reagents	Enzyme-catalyzed polymerization	[33]
Design for degradation	Enable safe degradation	Biodegradable pharmaceutical polymers	[34]
Real-time analysis	Preventing pollution during synthesis	Process analytical technology (PAT)	[35]
Inherently safer chemistry	Reduce accident risks	Mild reaction conditions	[4]

**Table 3:** Representative comparison of microwave-assisted and ionic liquid/deep eutectic solvent approaches versus conventional processing.

Approach	Representative System	Yield/Recovery	Purity/Quality Indicator	Time	Compared with Conventional	References
Microwave-assisted processing	Alginate–chitosan hydrogel synthesis	NR	Structurally confirmed by FTIR/DSC/SEM	2.5 min	Conventional heating typically requires longer bulk heating	[56]
Microwave-assisted extraction	Chitosan from Agaricus bisporus waste	NR	Degree of deacetylation 79.94%; solubility 75%	8 min deproteinization + 8 min deacetylation	Conventional extraction is described as slower and more chemically intensive	[57]
Microwave-assisted extraction (older direct comparison)	Chitosan from Rhizopus oryzae biomass	13.43% vs. 6.67% conventional	Degree of deacetylation 94.6 ± 0.9% vs. 90.6 ± 0.5% conventional	Shorter than conventional heating	Directly outperformed conventional extraction in yield and DD	[58]
NADES/DES extraction	Chitin/chitosan extraction from mushroom biomass	NR	Chitin 98.58%, chitosan 98.69% purity; DD up to 94.22%	NR	Reported to surpass traditional chemical extraction in purity	[59]
DES-based extraction (reviewed comparison)	Chitosan extraction	20–30% conventional vs. higher DES-dependent performance	Improved purity and lower harsh-chemical demand	Often shorter/milder, system-dependent	DES methods reduce chemical severity and can improve extraction efficiency	[60]

**Table 4:** Comparative analysis of conventional and green polymers in pharmaceutical manufacturing, highlighting differences in carbon emissions, energy consumption, and environmental impact. Green polymers demonstrate reduced carbon footprint, lower energy requirements due to mild synthesis conditions, and improved environmental sustainability compared to conventional petrochemical-based polymers.

Parameter	Conventional Polymers	Green Polymers	References
CO_2_ emissions	High (≈2–6 kg CO_2_/kg polymer)	Lower (≈0.5–2 kg CO_2_/kg polymer)	[63]
Energy consumption	High (petrochemical processing, high temp)	Reduced (enzymatic, microwave, mild conditions)	[64]
Environmental impact	Persistent pollution + microplastics	Reduced footprint + circular potential	[64]

## Data Availability

No new data were created or analyzed in this study.
